# Enhanced flexible supercapacitors with boron-doped graphene electrodes and carbon quantum dot gel electrolytes†

**DOI:** 10.1039/d4ra06990k

**Published:** 2025-02-14

**Authors:** Dilara Koroglu, Haluk Bingol, Betul Uralcan

**Affiliations:** a Department of Chemical Engineering, Bogazici University Bebek Istanbul 34342 Turkey betul.uralcan@boun.edu.tr +90(212)359-6871; b Science and Technology Research and Application Center (BITAM), Necmettin Erbakan University Konya 42090 Turkey; c Department of Basic Science, Faculty of Engineering, Necmettin Erbakan University Konya 42090 Turkey

## Abstract

Flexible solid state supercapacitors have gained significant importance in energy storage device technology. In this work, flexible solid-state supercapacitors are designed with enhanced capacitance, bending cycle stability and energy density. Activated carbon (AC) is synthesized from cabbage leaves and boron doped reduced graphene oxide (BRGO) is incorporated into AC to improve mechanical flexibility. On the other hand, carbon quantum dots (CQDs) and acetonitrile (ACN) as solvent are incorporated into a gel electrolyte. We investigate the concentration of boron in the electrode material and that of CQDs in the gel electrolyte and reveal that the capacitance, bending properties and energy density of the solid-state supercapacitor are simultaneously improved with the optimum composition of AC/BRGO in the CQD/gel electrolyte. This demonstration of composite electrode and electrolyte materials could substantially improve the capacitance, cycle stability and energy density of solid-state supercapacitors.

## Abstract 2

Flexible solid-state supercapacitors are designed with enhanced capacitance, bending cycle stability and energy density.

## Graphical abstract

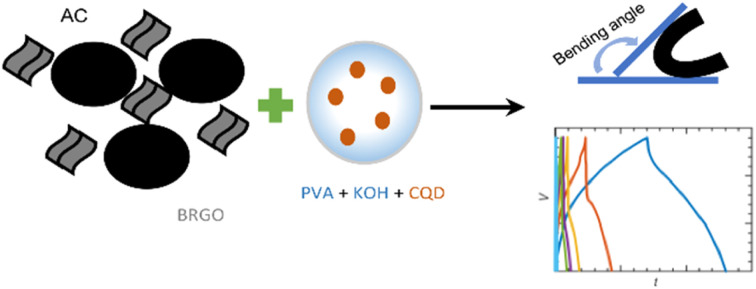

## 1 Introduction

As global energy demands rise with population growth, research efforts are increasingly directed towards optimizing energy storage devices. Among these, flexible solid-state supercapacitors stand out for their excellent bending properties, cycling stability, and high power density. Obtaining enhanced performance in these devices relies on the rigorous selection and design of electrode and electrolyte materials.^1–8^

Activated carbons (ACs) are widely used in supercapacitors due to their large surface area, high porosity, commercial availability, and cost-effectiveness.^9^ ACs are typically produced through physical or chemical activation processes, offering versatility for various applications.^10^ However, ACs tend to be brittle, necessitating the incorporation of carbon additives to enhance flexibility while maintaining cycling stability.^11^ Among these additives, reduced graphene oxide (RGO) stands out for its tunable flexibility and electrochemical stability.^11–13^ Recent advancements have seen RGO being doped with heteroatoms like boron, nitrogen, phosphorous, and sulfur, enhancing its electronic properties.^14–17^

Boron doping introduces boron atoms into the graphene lattice, replacing carbon atoms. This substitution can lead to lattice distortions, potentially affecting the material's mechanical properties. However, the strong boron–carbon bonds can help maintain structural integrity, and the specific impact on mechanical properties depends on factors such as doping concentration and distribution.^18^ Incorporating boron-doped reduced graphene oxide (BRGO) into an activated carbon (AC) matrix can enhance the composite's mechanical stability. The interaction between BRGO sheets and the AC matrix facilitates stress distribution during mechanical loading, reducing the likelihood of fracture. Additionally, boron doping can improve the adhesion between graphene sheets and the matrix, further enhancing mechanical stability. For example, Wang *et al.* demonstrated that boron doping at high concentrations (>1%) could decrease the material's tensile strength by inducing defects^19^*via* the molecular dynamic (MD) simulations. Dai *et al.* reported that graphene can maintain a large fraction of its pristine strength and stiffness after substituting boron for carbon atoms by MD simulations.^20^ Further, Peng *et al.* explored boron-doped porous graphene as an electrode material for flexible devices, achieving three times the areal capacitance compared to non-doped counterparts and maintaining 90% capacitance retention over 10 000 cycles under bending conditions.^21^ Similarly, Pandian *et al.*^18^ synthesized boron-doped reduced graphene for flexible solid-state supercapacitors, reporting a capacitance of 266 F g^−1^ at a current density of 1 A g^−1^ and 98% capacitance retention over 5000 cycles at 5 A g^−1^. Addition to those studies, boron doping leads to a significant improvement in the material's capacitance, which is a critical property for supercapacitors and energy storage devices. For example, Niu *et al.* reported that 4% boron-doped graphene exhibited an 86% improvement in capacitance compared to pristine graphene.^22^ Similarly, Zuo *et al.* observed a gravimetric capacitance of up to 281 F g^−1^ for porous boron-doped graphene F g^−1^.^23^ Han *et al.* demonstrated a capacitance of 200 F g^−1^ for BRGO in 6 M KOH,^24^ while Yeom *et al.* reported an outstanding capacitance of 448 F g^−1^ for BRGO under the same conditions.^25^ Thirumal *et al.* observed a capacitance increase from 53 F g^−1^ to 113 F g^−1^ upon boron doping of graphene oxide.^26^ Boron, being electron-deficient, introduces holes into the graphene structure, enhancing hole concentration and facilitating charge transport.^27^ This p-doping effect improves electrical conductivity, which, combined with the mechanical properties, makes BRGO a promising material for energy storage applications.^28^

Electrolytes also play a crucial role in determining the energy density and safety of supercapacitors.^29–35^ Gel polymer electrolytes (GPEs) have emerged as key components in solid-state supercapacitors due to their high conductivity and enhanced flexibility.^36,37^ These GPEs serve dual roles as both electrolyte and separator in flexible supercapacitor configurations.^38^ Optimizing GPE performance involves selecting the appropriate combination of host polymer, solvent, and electrolytic salt. Poly(vinyl alcohol) (PVA) is widely used for preparing gel polymer electrolytes, owing to its high hydrophilicity, affordability, and safety.^39^ The preparation of gel electrolytes involves mixing PVA with various aqueous solutions of electrolytic salts, such as H_2_SO_4_, H_3_PO_4_, KOH, NaOH, KCl, NaCl, and LiCl, to facilitate charge transfer and improve capacitance.^40^ Since water is used to dissolve PVA/KOH mixture, potential window is restricted to 1 V. Herein, we incorporate acetonitrile (ACN) into water as solvent for the gel polymer electrolyte to widen the operating potential window; thereby, enhancing energy density. The incorporation of ACN into water for gel electrolyte preparation represents a novel approach in the literature. Previous studies have explored various additives in gel polymer electrolytes. For instance, Guofu and colleagues demonstrated that the addition of K_3_Fe(CN)_6_ to PVA/KOH gel electrolyte improved specific capacitance to 431 F g^−1^.^41^ Haijun *et al.*^42^ investigated KI incorporation into PVA/KOH gel electrolyte with activated carbon electrodes, achieving a specific capacitance of 237 F g^−1^.^42^ As compared to other carbon materials, CQDs have ultra small sizes (less than 10 nm) and abundant functional groups on their surface, which donate them with uniform dispersion and excellent electron transfer/reservoir properties.^43,44^ The incorporation of CQDs into the pores of activated carbon was explored by Kumar *et al.* and this electrode material showed high capacitance and very stable electrochemical behavior.^45^ Kumar *et al.* explored the performance of CQDs as aqueous electrolytes, achieving a specific capacitance of 155 F g^−1^ for graphene-based electrodes.^46^ Herein, carbon quantum dots were chosen to be added to the electrolyte due to their unique properties. Their incorporation can increase the overall performance by facilitating better ion transport, improving the interface between the electrolyte and electrode leading to better charge transfer, contributing to the stability of the device. CQDS with exceptional conducting properties arise from the quantum confinement and edge effect, which is related to the motion of charge carriers (like electrons) in such small structures. The motion of charges becomes restricted in one or more dimensions, leading to discrete energy levels, which alters the electronic properties such as increased conductivity.^47,48^ Further, the presence of diverse heteroatom-containing functional groups (such as oxygen and nitrogen) on carbon nanomaterials provides numerous active sites that improve electrochemical performance.^46,49^

The rest of this paper is organized as follows. In Section 2, we describe the material synthesis process, device fabrication and give details on structural and electrochemical characterization techniques. In Section 3, we report the structural and electrochemical characterization results, and discuss our findings on the fabricated solid-state supercapacitors. In Section 4, we provide concluding remarks and suggest some possible directions for future inquiry.

## 2 Experimental section

### 2.1 Synthesis of activated carbon from cabbage leaves

Cabbage leaves (4 g) were cut, washed, and dehydrated at 110 °C for 12 hours. The dehydrated cabbage leaves were carbonized at 600 °C for 2 hours in a tubular furnace (Protherm) under argon (Ar) protection. Subsequently, the carbonized cabbage leaves underwent chemical activation with potassium hydroxide (KOH, Merck–pellets for analysis) solution (Cabbage leaves : KOH ratio of 1 : 4) at room temperature for 10 hours on a magnetic stirrer. The resulting solution was dried at 80 °C and annealed at 800 °C (with a heating rate of 5 °C min^−1^) for 2 hours under Ar atmosphere. Finally, the products were washed with 1 M hydrochloric acid (HCl, Merck, 37% for anaylsis) and distilled water *via* centrifugation (1500 rpm, 20 min, 25 cycles) until the solution was neutral. The resulting solution was dried at 80 °C overnight to obtain activated carbon (AC). The overall AC production procedure is given in our previous work.^50^

### 2.2 Preparation of CQD

CQDs were synthesized using citric acid (Sigma-Aldrich) and urea (Sigma-Aldrich) *via* solvothermal processes, as depicted in our previous work.^50^ Briefly, 0.384 g citric acid and 0.060 g urea were dissolved in 10 mL dimethylformamide (DMF, Sigma-Aldrich) and then sonicated to ensure uniform mixing. The mixture underwent a solvothermal reaction at 180 °C for 6 h in a teflon-lined stainless autoclave (50 mL). The resulting brown solution was purified using a dialysis bag (MWCO: 0.5 kDa, Spectrum Laboratories Inc. (USA)) for 15 minutes. Ultra-pure water were freshly prepared using a Millipore Direct-Q3 water purification system. The obtained CQD solution was stored at +4 °C.

### 2.3 Structural characterization

(N_2_) adsorption–desorption analyses were conducted using a Micromeritics ASAP 2020 Plus Chemi automated instrument to determine the micropore structure of the samples (AC and AC-10BRGO). The materials were degassed at 240 °C for 6 hours and the specific surface area was calculated using the Brauner Emmett Teller (BET) method. Raman analysis was performed on powder AC and AC-10BRGO (Reinshaw inVia, *λ* = 532 nm). Surface chemistry was investigated using X-ray photoelectron spectroscopy (XPS) with a Thermo KAlpha X-ray Photoelectron Spectrometer. The gel electrolytes were characterized by thermal gravimetric analysis (TGA) using a TA Instruments SDT 650, X-ray diffraction (XRD) using a PANalytical X'Pert PRO with a wavelength of *λ* = 1.54059 Å, and Fourier-transform infrared (FTIR) spectroscopy using a Bruker Vertex 80v spectrometer.

### 2.4 Fabrication and characterization of solid-state supercapacitors

#### 2.4.1 Preparation of the gel electrolyte

Gel polymer electrolyte films were prepared *via* the solutioncasting method. A mixture of 30% acetonitrile (ACN) and 70% water was prepared. Subsequently, 1 g of KOH and 1 g of polyvinyl alcohol (PVA, Merck in powder form) were dissolved separately each in 25 mL of the ACN-water solution and mixed using a magnetic stirrer at 95 °C for 3 hours. The two solutions were then combined and stirred vigorously for 15 minutes to form a homogeneous mixture. CQDs, dissolved in dimethylformamide (DMF) as 5 mg mL^−1^, were added to the PVA-KOH/ACN-water gel electrolyte at varying concentrations (0.5%, 1%, and 1.5% by weight). The mixture was stirred at 70 °C for 3 hours to ensure uniform dispersion. Finally, the homogeneous solution was cast into a glass Petri-dish and dried at 95 °C for ≃1 hour to form the gel polymer electrolyte films.

#### 2.4.2 Preparation of the electrodes

At first, AC was mixed with BRGO (Graphitene) in an agate mortar. This composite material was then physically blended with acetylene black (AB, Nanografi) and polytetrafluoroethylene (PTFE, Nanografi) at a weight ratio of 8 : 1 : 1. *N*-Methyl-2-pyrrolidone (NMP) was added to the mixture at a ratio of 0.15 g mL^−1^, followed by mixing at 300 rpm for 2 hours at 70 °C. The obtained slurry was further stirred using a magnetic stirrer at room temperature for 12 hours to ensure homogeneity. Silver nanoparticles ink (Sigma-Aldrich), was printed on a flexible polyethylene terephthalate (PET) film with a surface area of 1 cm^2^ and heated at 130 °C for 5 hours. The AC/BRGO/PTFE slurry was uniformly spread onto the silver current collector using a doctor blade and dried at 95 °C for 1 hour.

#### 2.4.3 2-Electrode electrochemical measurements

The flexible solid state supercapacitor was assembled as described here. The gel film electrolyte was sandwiched between two electrodes. The mass of each electrode in our symmetric supercapacitor is 1 mg ± 0.05 mg. To ensure optimal contact between layers, the assembled supercapacitor was subjected to heating at 95 °C for 3 min. Electrochemical characterization was conducted using a BioLogic SP300 potentiostat instrument employing the standard 2-electrode set-up (Fig. S1†).

## 3 Results and discussion

### 3.1 Structural characterization of active electrode and electrolyte materials

#### 3.1.1 Cabbage-derived activated carbon/boron doped reduced graphene oxide

The structural properties of the activated carbon (AC) and AC-10BRGO composite were characterized using nitrogen (N_2_) adsorption–desorption isotherms, pore size distribution analysis, X-ray photoelectron spectroscopy (XPS), Raman spectroscopy, Fourier-transform infrared (FTIR) spectroscopy, and X-ray diffraction (XRD).

**Fig. 1:**
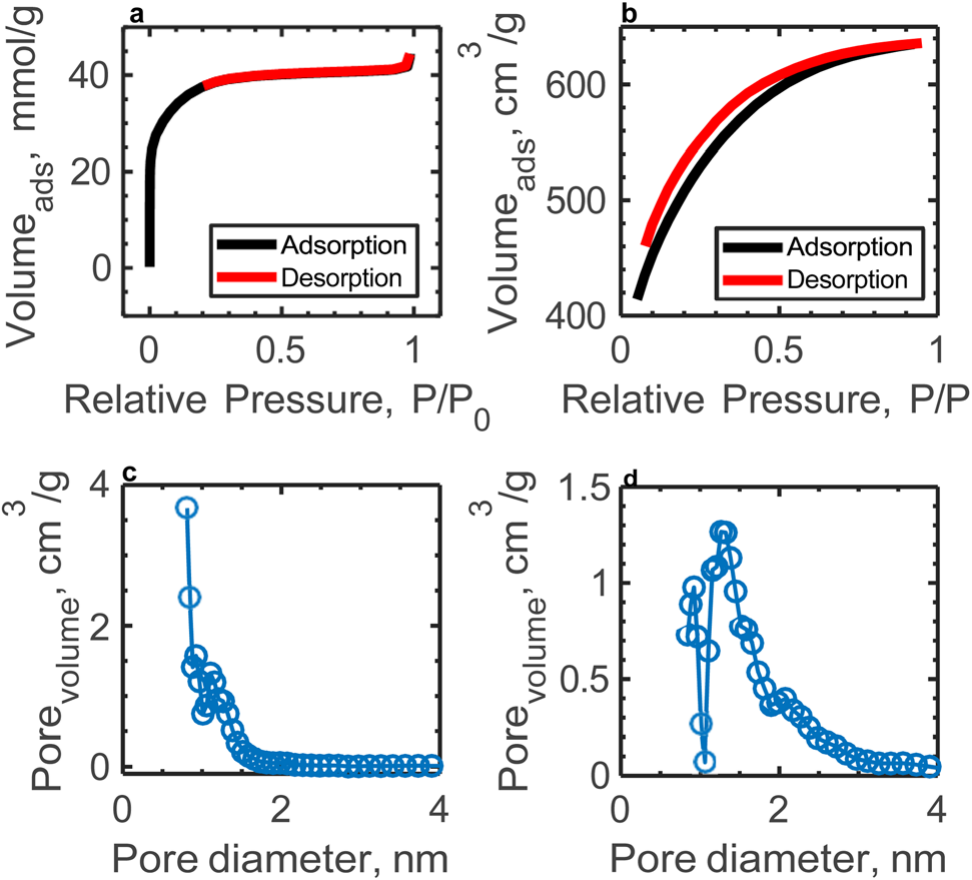
Pore structure analysis of AC and AC-10BRflO (a) N_2_ adsorption/desorption isotherms of AC, (b) N_2_ adsorption/desorption isotherms of AC-10BRflO, (c) pore size distribution of AC, and (d) pore size distribution of AC-10BRflO.

Fig. 1a and b display the N_2_ adsorption–desorption isotherms for AC and AC-10BRGO, respectively. The AC exhibits a type I isotherm characteristic of a microporous structure dominated by micropores, as per the IUPAC classification. In contrast, AC-10BRGO demonstrates a hybrid type I–IV isotherm, indicating a bimodal micro/mesoporous pore structure. The nitrogen is adsorbed by the AC-10BRGO, mainly, at low relative pressures which is typical of microporous structure. At higher relative pressures, with continued adsorption, mesoporosity is present in the structure of AC-10BRGO. The pore size distribution curves, shown in Fig. 1c and d for AC and AC-10BRGO respectively, further elucidate these findings. The specific surface area and cumulative pore volume of AC are measured as 3038 m^2^ g^−1^ and 1.02 cm^3^ g^−1^, respectively, while AC-10BRGO possesses a smaller BET surface area (1724 m^2^ g^−1^) and cumulative pore volume (0.98 cm^3^ g^−1^).

**Fig. 2:**
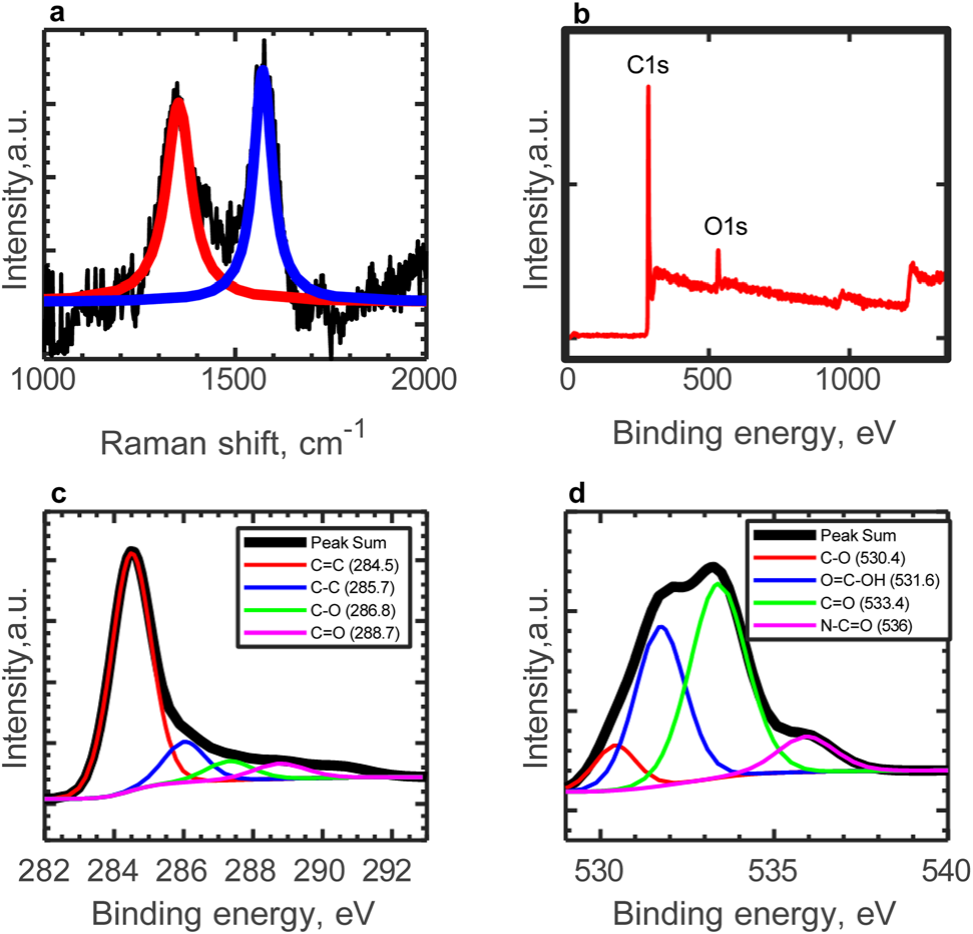
Characterization of AC (a) Raman spectroscopy analysis of AC, fitting curves. Red line represents the Lorentzian fit to the D-peak and blue line represents the BWF fit to the fl-peak, (b)the total XPS spectra, (c) deconvoluted C 1s XPS Spectra, and (d) deconvoluted O 1s XPS Spectra.^50^

The Raman and XPS analyses of AC were given in our previous work.^50^ The Raman spectrum of AC reveals D- and G-peaks centered at 1360 and 1570 cm^−1^, respectively (Fig. 2a).^50^ These peaks are fit using the Lorentzian and Breit–Wigner–Fano functions for D- and G-peaks, respectively. The D-band is attributed to the defect of highly ordered carbonaceous materials while the G-band is at-tributed to C=C strecthing vibrations. The peak intensity ratio of the D-band and the G-band indicates the carbon-containing defects. The ratio of the intensities of the peaks (ID/IG) is obtained as 0.92, indicating a defective nature of AC.

XPS spectra of AC, depicted in Fig. 2b, depict a C/O ratio of ≃11.^50^ To further investigate the oxygen functional groups on the surface of AC, the deconvoluted C 1s and O 1s XPS spectra are presented in Fig. 2c and d, respectively.^50^ The C 1s peak is deconvoluted into four peaks, indicating various carbon functionalities. Meanwhile, the O 1s spectra suggest that C=O components dominate the surface functionalities of AC, followed by carboxylic functional groups (O=C–OH). The presence of N_2_ doping into the carbon structure is confirmed by the peak at 536.0 eV.

**Fig. 3:**
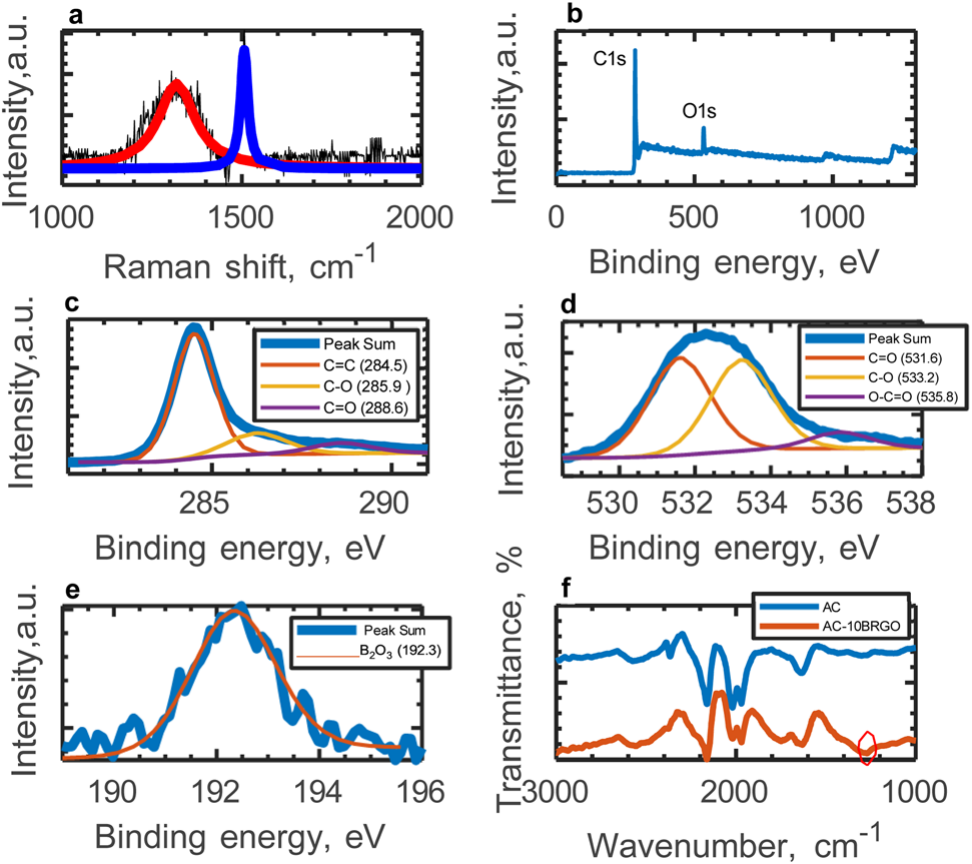
Characterization of AC-10BRflO (a) Raman spectroscopy analysis of AC-10BRflO. Red line represents the Lorentzian fit to the D-peak and blue line represents the BWF fit to the fl-peak, (b) the total XPS spectra, (c) deconvoluted C 1s XPS Spectra, (d) deconvoluted O 1s XPS Spectra, (e) deconvoluted B 1s XPS Spectra, and (f) FTIR analysis of AC and AC-10BRflO.

The composite material, prepared by incorporating BRGO into AC, was also characterized by Raman, XPS, and FTIR. Raman spectra of RGO and BRGO show that the ratio of the D-band to G-band (ID/IG) in the Raman spectra of the RGO samples is 1.1 and that of BRGO is 2.2 (Fig. S2†). ID/IG ratio of 1.1 indicates a relatively moderate defect density in RGO. This suggests that a significant amount of oxygenated functional groups have been removed and some of the graphitic structure have been restored. The moderate ratio indicates that while the material is not pristine graphene, it has good potential for conductivity. On the other hand, ID/IG ratio for BRGO (2.2) indicates that BRGO has a significantly higher defect density compared to RGO. This may be attributed to additional defects or functional group modifications introduced into the graphene lattice during the synthesis of BRGO. Specifically, some reduction processes can lead to over-reduction, disrupting the graphene network and increasing disorder. The high level of disorder in the material creates defects that can act as active sites, enhancing electrochemical performance. Furthermore, the presence of additional oxygenated groups in the BRGO structure could improve electrolyte wettability, contributing to its overall performance. This relatively high ID/IG ratio is consistent with the introduction of defects and functional groups, which is typically expected in the presence of dopants, such as boron. The ID/IG ratio of AC-10BRGO, determined from Raman spectroscopy (Fig. 3a), was found to be 3, much higher than that of AC (0.92), suggesting successful boron doping on the reduced graphene oxide sheet. This increase in the ID/IG ratio indicates the presence of defects, potentially acting as active sites, may have enhanced capacitance.

In order to further explore the type and composition of functional groups on the surface of AC-10BRGO, the deconvoluted C 1s and the O 1s XPS spectra are presented in Fig. (Fig. 3c and d). C 1s peak is deconvoluted into three peaks, *i.e.* C=C at 284.5 eV, C–O at 285.9 eV, and C=O at 288.6 eV. The O 1s peak is deconvoluted into three peaks, *i.e.* C=O at 531.6 eV, C–O at 533.2 eV and O–C=O at 535.8 eV, indicating oxygen surface functionalities that may improve the wettability of the electrodes. XPS analysis also confirms the presence of boron in the AC-10BRGO structure. A peak found at 192.3 eV is a characteristic of B–O bond in B_2_O_3_, which proves boron incorporation into AC structure.

Additionally, XRD patterns of AC and AC-10BRGO (Fig. S3†) display peaks at 24.5° and 42.8°, with AC-10BRGO showing more intense peaks, confirming successful boron doping. These findings are consistent with the results obtained from Raman, XPS, and FTIR analyses.

Scanning electron microscopy (SEM) was used to gather information on the morphology of the powdered activated carbon and activated carbon-boron doped reduced graphene oxide (Fig. S6†). The image (Fig. S6a†) shows irregularly shaped particles with rough and porous surfaces, characteristic of activated carbon. The particle size distribution from Fig. 1c appears heterogeneous, with some larger and smaller aggregates. The porous structure suggests a high surface area (Fig. 1c). The composite (Fig. S6b†) exhibits a layered and sheet-like morphology, typical of reduced graphene oxide (RGO). The boron doping may have caused defect formation and wrinkling, leading to the observed folded and stacked layers. The layers appear to be interconnected, which can enhance electrical conductivity and ion transport pathways. AC is not distinctly visible as separate particles but the layered morphology of the BRGO likely encapsulates or intercalates the AC particles. This can occur during the synthesis process, where AC becomes embedded in or coated by the BRGO sheets. AC may contribute to the overall roughness or slight irregularities seen in the BRGO layers, as it provides a porous and textured substrate for the graphene sheets to anchor onto. While the BRGO layers dominate the visual morphology, AC likely maintains its porous structure within the composite as depicted by Fig. 1c and d. These pores might not be directly visible due to the overlapping graphene layers but still contribute to the composite's overall surface area and ion accessibility. In this material, AC could serve as a structural backbone, providing mechanical stability to the composite. The layered BRGO sheets alone might be prone to agglomeration or collapse, but the dispersed AC particles may help maintain separation between layers and improve the structural integrity of the composite. While AC is not really visible, the wrinkles, folds, and roughness in the BRGO layers could be influenced by the underlying AC particles, indicating a potential interaction between the two materials. This interaction could also enhance the composite's conductivity and ion transport properties.

#### 3.1.2 The gel electrolytes

**Fig. 4:**
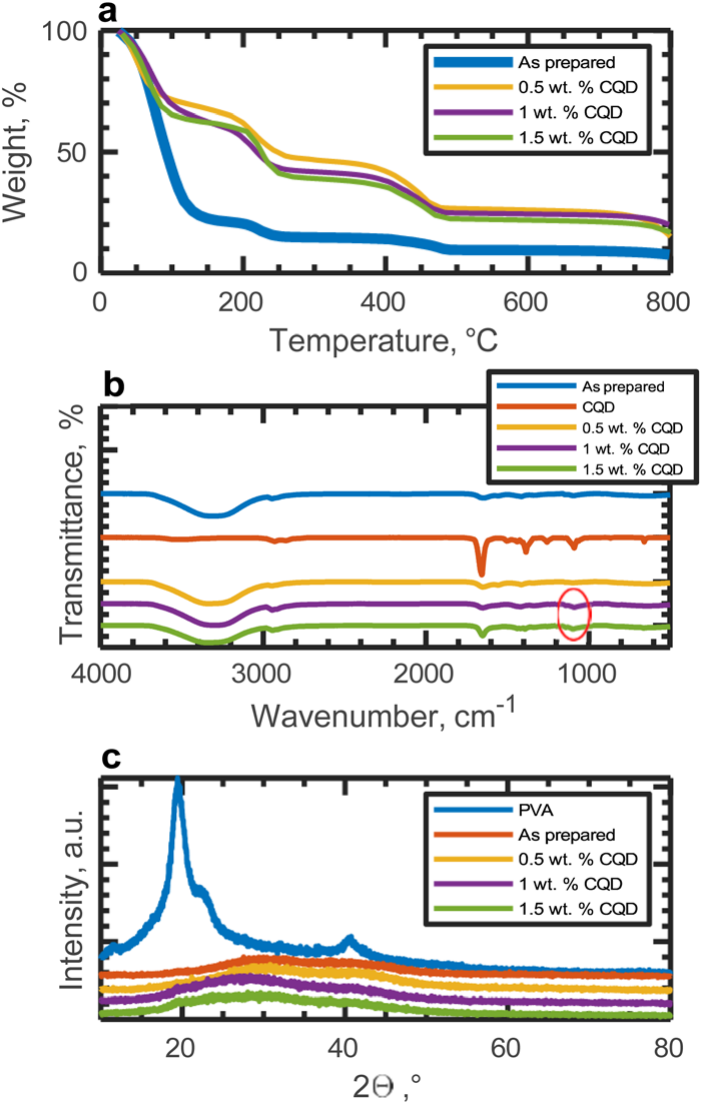
Structural characterizations of solo gel-electrolytes (a) TflA curves, (b) FTIR spectrum and (c) XRD spectrum.

The thermal stability of the gel electrolytes was evaluated through thermal-gravimetric analyses (TGA), and the obtained TGA curves are depicted in Fig. 4a. For the gel electrolytes, namely PVA-KOH and PVA-KOH-CQD, the initial mass loss occurs around 105 °C. A subsequent weight loss is observed at 211 °C, attributed to the elimination of OH groups and side chains. A third weight loss is noted around 485 °C, corresponding to the breaking of the PVA backbone. ^51^ Notably, complete decomposition of PVA-KOH is observed at 800 °C; however, the addition of CQDs enhances thermal stability, with 20% of the 1 wt% CQD-added gel electrolyte remaining at 800 °C. Details on the structural characterization of CQDs can be found at our previous work.^50^

FTIR spectra were obtained for CQD, as well as as-prepared and CQD-added gel electrolyte samples. The presence of bands at 3330 and 2900 cm^−1^ is attributed to O–H stretching and bending, and CH_2_ asymmetric stretching, respectively. ^51^ Additionally, bands at 1650 and 1100 cm^−1^ (as indicated by the red circle in Fig. 4b) correspond to C=O and C–O stretching, respectively, indicating the incorporation of CQDs into the gel electrolyte.

XRD patterns of pure PVA, as-prepared and CQD-added gel electrolyte films are illustrated in Fig. 4c. The XRD spectrum of PVA exhibits a peak at 20°, indicative of a semi-crystalline structure. ^52^ However, in the prepared gel electrolyte, addition of KOH to PVA results in an amorphous structure. Upon incorporation of CQDs, a broad peak is observed in the range of 20–40°, suggesting an increase in the amorphous domain of the material. ^53^ Although the mechanism of ion transportation in gel electrolytes remains unclear, it is speculated that polymer chain motion exists within the amorphous structure. ^51,53^

### 3.2 Electrochemical characterization

#### 3.2.1 Performance of cabbage-derived activated carbon/boron doped reduced graphene oxide composites

Cyclic voltammetry measurements were conducted at different scan rates for varying concentrations of boron-doped reduced graphene oxide (AC-5BRGO, AC-10BRGO, AC-15BRGO) using 30% v/v ACN during gel electrolyte preparation. Rational design of the layered structure of BRGO in AC can effectively prevent the restacking of graphene sheets, enhancing the usage of surface area and establish effective ion transport channels. ^54^ The resulting cyclic voltammograms are presented in Fig. 4a–d. Capacitance was calculated from the cyclic voltammetry cycles using the equation1
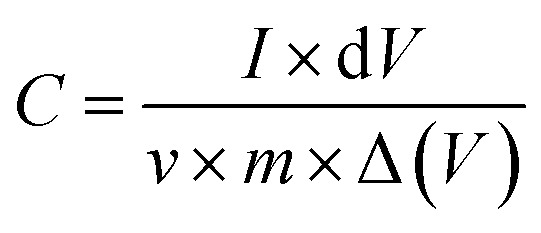
where *I* is the current, *V* is the applied potential, *v* is scan rate, and *m* is the mass of the electrode material.

**Fig. 5:**
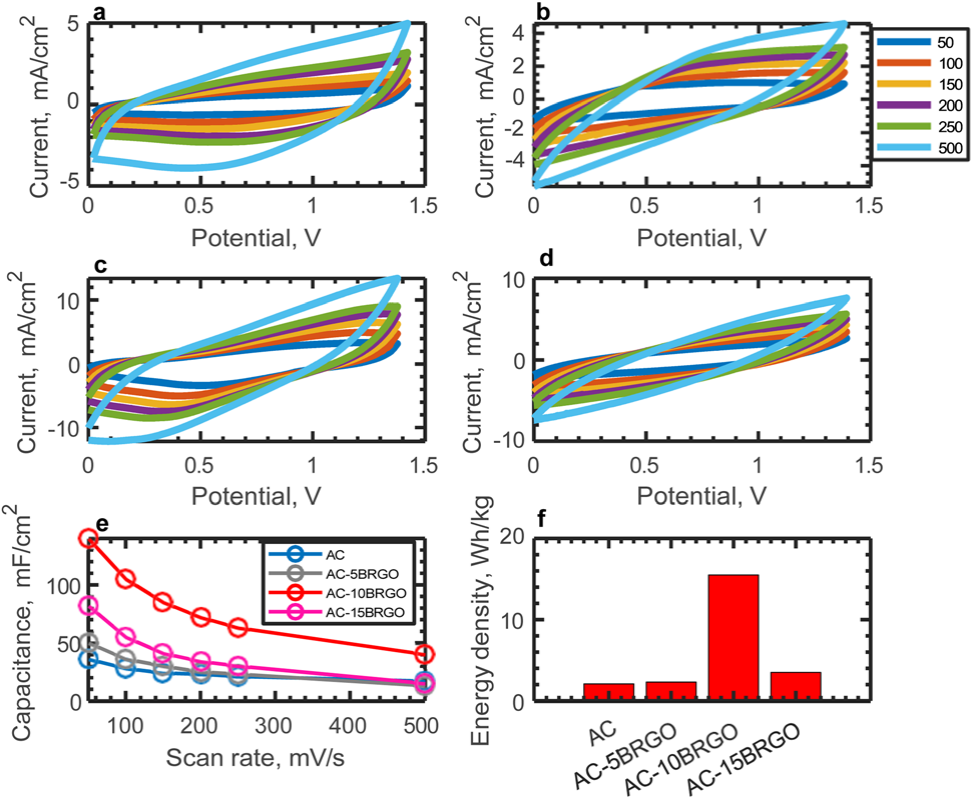
Electrochemical characterizations of AC and AC-BRflOs (a) cyclic voltammograms of AC at different scan rates, (b) cyclic voltammograms of AC-5BRflO at different scan rates, (c) cyclic voltammograms of AC-10BRflO at different scan rates, (d) cyclic voltammograms of AC-15BRflO at different scan rates, (e) capacitance *vs.* scan rate of electrode materials and (f) energy densities of the electrode materials at 50 mV s^−1^ at 1.4 V potential window.

The electrochemical measurements conducted at various boron concentrations (AC-5BRGO, AC-10BRGO, AC-15BRGO) provide insights into the performance of the electrodes. Fig. 5a–d depict the cyclic voltammograms of the samples at scan rates ranging from 50 to 500 mV s^−1^ within a 1.4 V potential window, demonstrating their electrochemical stability. Furthermore, Fig. 5e presents the specific capacitance as a function of scan rate for each boron concentration. The optimal boron concentration is determined to be 10 wt%, resulting in an areal capacitance of 140 mF cm^−2^ at 50 mV s^−1^. The comparison of structural character-izations of AC and AC-10BRGO are given in Section 3.1. Based on N_2_ adsorption–desorption isotherms, both AC and AC-10BRGO has a microporous structure. The pore size distribution of AC is centered at 0.74 nm and that of AC-10BRGO is centered at 1.3 nm. The specific surface area of AC-10BRGO (1724 m^2^ g^−1^) is smaller than that of AC (3038 m^2^ g^−1^), suggesting that although specific surface area is significant for ion adsorption, the accessibility of electrolyte ions to the electrode surface outweighs for a better electrochemical performance.^55^ The defective structure observed in AC-10BRGO, indicated by the threefold higher ID/IG ratio (3) compared to AC (0.92) from Raman analysis, suggests a significant role in ion adsorption due to the presence of defects, serving as active sites for electrolyte ions.^56,57^ Further confirmation is provided by XPS spectra, revealing a dominance of oxygen surface functionalities in AC-10BRGO, as evidenced by the lower C/O ratio (8.96) compared to AC (11). The enhanced wettability resulting from the decoration of oxygen functional groups^58^ on the electrode surface is supported by both Raman and XPS analyses. Additionally, FTIR results corroborate these findings, suggesting successful boron doping into the AC structure due to B–O bond at 1300 cm^−1^ and the presence of oxygen functionalities on the electrode surface.

#### 3.2.2 Performance of symmetric supercapacitors with CQD incorporated gel electrolyte

We integrated BRGO into AC structure during electrode preparation in order to improve charge transfer and elevate ion accessibility. BRGO amount in AC porous network was optimized as 10 wt% (AC-10BRGO). AC-10BRGO was further tested with CQD contents of 0.5 wt%, 1 wt%, 1.5 wt% in the gel polymer electrolyte.

**Fig. 6:**
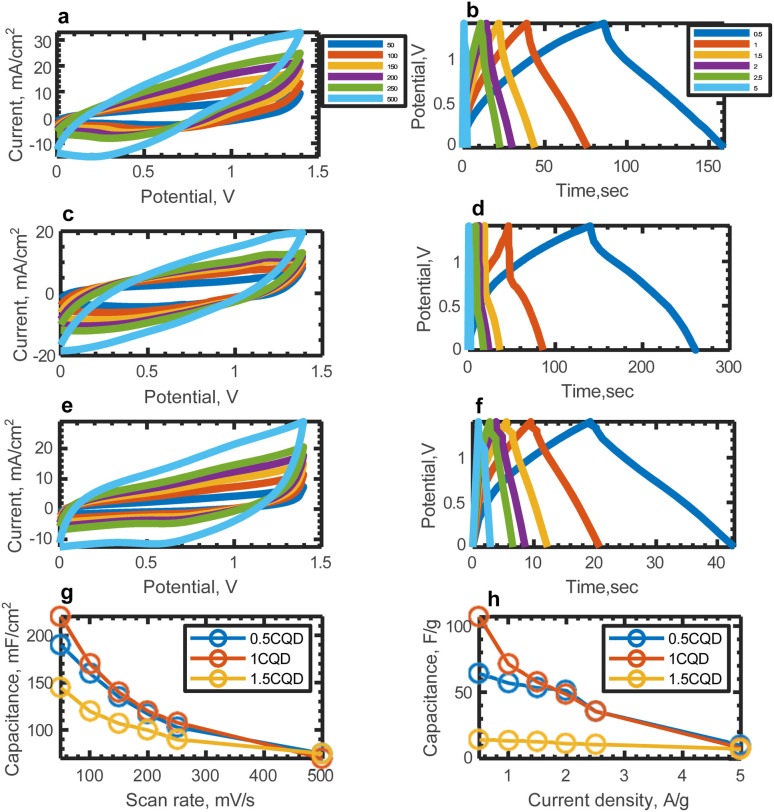
Electrochemical characterizations of AC-10BRflO with the CQD contents of 0.5 wt%, 1 wt%, 1.5 wt% in gel electrolyte at various scan rates/current densities (a) CV of 0.5CQD, (b) flCD curves of 0.5CQD, (c) CV of 1CQD, (d) flCD curves of 1CQD, (e) CV of 1.5CQD, (f) flCD curves of 1.5CQD, (g) A real capacitance *vs.* scan rate of materials, (h) capacitance *vs.* current density of materials, (i) energy densities of the materials at 50 mV s^−1^ at 1.4 V potential window and (j) comparison of energy densities with literature.

Fig. 6a, c and e display the cyclic voltammograms of the materials at various scan rates ranging from 50 to 500 mV s^−1^. Additionally, charge–discharge curves spanning from 0.5 to 5 A g^−1^ are depicted in Fig. 6b, d and f.

The capacitance from charging–discharging cycle is calculated as a function of current density using2
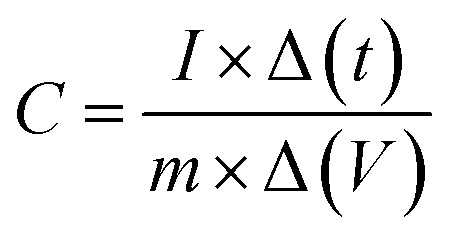
where *I* is the discharged current, Δ*t* is the discharge time, *m* is the mass of the electrode material, and Δ*V* is the voltage change. Areal and mass capacitances are compared in Fig. 6g and h for different loading of CQD in gel electrolyte.

Cyclic voltammograms and GCD curves indicate that charge storage is primarily through physical adsorption of ions. 1.5 wt% CQD addition to gel electrolyte has an adverse impact on capacitance (14.2 F g^−1^ at 0.5 A g^−1^) and one plausible explanation for the reduced capacitance at the highest CQD concentration is that CQDs may agglomerate and cause blockage within polymeric network. On the other hand, 1 wt% CQD loading in gel polymer electrolyte enhances capacitance (107 F g^−1^ at 0.5 A g^−1^), energy density (29 W h kg^−1^ at 1.4 V) and scan rate dependence, which may be attributed to the fast charging properties of the composite materials. This composite material exhibits enhanced energy density performance compared to the metrics reported by other researchers for symmetric solid-state supercapacitors (Table S1†). The electrochemical characterizations of CQD added gel electrolyte confirm the results obtained from the structural characterizations which indicate the successful incorporation of CQD into gel electrolyte, as discussed in Section 3.1.2.

Ionic conductivity can be calculated by the equation below:3
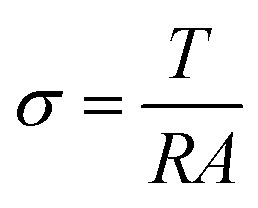
where *T* is the thickness of the film (cm), *R* is the bulk resistance (Ω) obtained from the first intercept on the *x*-axis of the impedance data in the complex plane, and *A* is the contact area (cm^2^). The thickness of the as-prepared gel film is measured to be 64 μm, while that of the CQD-incorporated gel film is 128 μm. With a contact area of 1 cm^2^, the resistance is determined from impedance results (Fig. S5†). Using this information, the ionic conductivity of the as-prepared gel film is calculated as 0.40 mS cm^−1^, compared to 1.94 mS cm^−1^ for the CQD-incorporated gel film. The incorporation of carbon quantum dots (CQDs) significantly enhances the ionic conductivity of the gel electrolyte, likely due to the improved charge carrier mobility provided by the CQDs, which facilitates more efficient ion transport within the gel matrix.

#### 3.2.3 Flexibility of assembled symmetric solid state supercapacitors

**Fig. 7:**
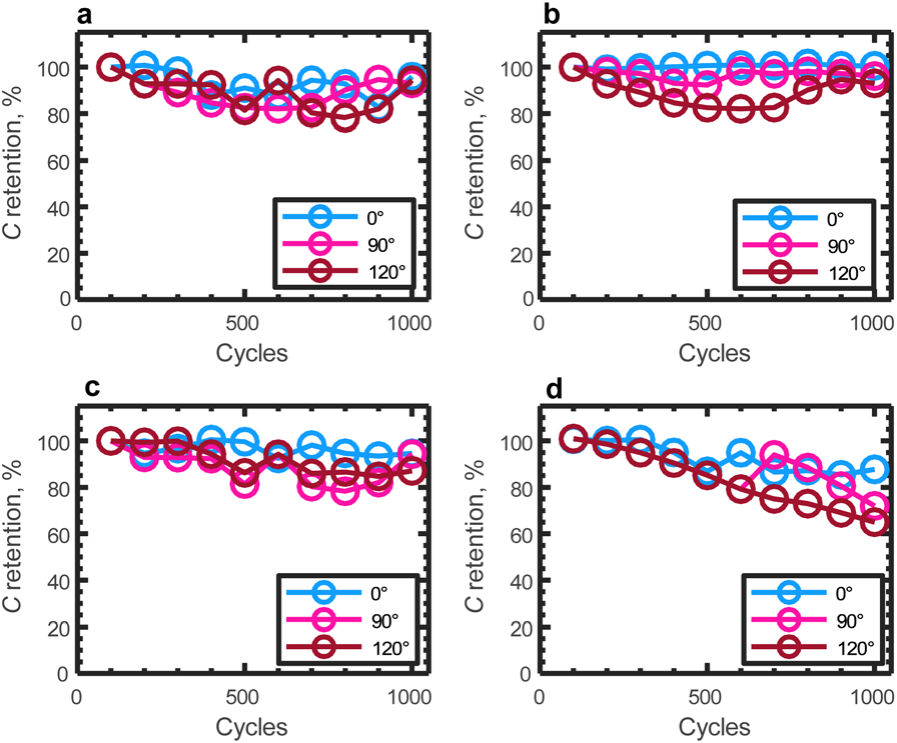
Capacitance retentions at 5 A g^−1^ over 1000 cycles under different bending angles of (a) AC-10BRflO, (b) AC-0.5CQD, (c) AC-1CQD and (d) AC-1.5CQD.

To evaluate the flexibility of the devices, they were subjected to bending at various angles (90° and 120°), and capacitance retention was measured over 1000 cycles at a current density of 5 A g^−1^. Fig. 7 illustrates the capacitance retention under bending angles of 0°, 90° and 120°. It is evident that the structural integrity of AC-10BRGO, 0.5CQD, and 1CQD remains intact when folded, with capacitance retention exceeding 90% over 1000 cycles under various bending angles, indicating excellent flexibility. However, 1.5CQD exhibits inferior performance compared to other materials, possibly due to CQD coagulation when folded.

## 4 Conclusion

In this study, we have successfully fabricated flexible solid-state supercapacitors using cabbage-derived activated carbon (AC) and boron-doped reduced graphene oxide (BRGO) composites. We have elucidated the structural and electrochemical properties of the materials using structural and electrochemical characterization techniques including N_2_ adsorption–desorption measurements, XPS, Raman spectroscopy, FTIR spectroscopy and cyclic voltammetry.

The electrochemical measurements conducted at various boron concentrations (AC-5BRGO, AC-10BRGO, AC-15BRGO) provided insights into the performance of the electrodes. Specifically, AC-10BRGO exhibited superior electrochemical stability and specific capacitance, with an optimal boron concentration of 10 wt%. Moreover, the flexibility of the assembled supercapacitors was evaluated by subjecting them to bending at different angles (90° and 120°). AC-10BRGO, 0.5CQD, and 1CQD exhibited excellent flexibility, maintaining structural integrity with capacitance retention exceeding 90% over 1000 cycles under various bending angles. Overall, our findings highlight the promising potential of cabbage-derived AC and BRGO composites for flexible solid-state supercapacitor applications. The combination of superior electrochemical performance and flexibility makes these materials attractive candidates for various portable and wearable electronics, energy storage systems, and flexible electronic devices. Further optimization and exploration of these materials could lead to significant advancements in the field of flexible energy storage technologies.

## Data availability

The data supporting this article have been included as part of the ESI.†

## Author contributions

Dilara Koroglu: investigation, validation, data curation, writing – review and editing. Haluk Bingöl: resources. Betul Uralcan: conceptualization, resources, supervision, funding acquisition.

## Conflicts of interest

There are no conflicts to declare.

## Supplementary Material

RA-015-D4RA06990K-s001

RA-015-D4RA06990K-s002

RA-015-D4RA06990K-s003

RA-015-D4RA06990K-s004

RA-015-D4RA06990K-s005

RA-015-D4RA06990K-s006

RA-015-D4RA06990K-s007
