# Supplementary material for: Enhanced flexible supercapacitors with boron-doped graphene electrodes and carbon quantum dot gel electrolytes
Source: RSC Adv. 2025 Feb 14;15(7):5011–9. doi: 10.1039/d4ra06990k (PMC11827676; doi:10.1039/d4ra06990k)
Supplement: RA-015-D4RA06990K-s001 [file RA-015-D4RA06990K-s001.pdf]

Supplementary Information (SI) for RSC Advances.
This journal is © The Royal Society of Chemistry 2025

Enhanced Flexible Supercapacitors with Boron-Doped Graphene Electrodes and Carbon Quantum Dot Gel Electrolytes

Dilara Koroglu[a], Haluk Bingöl[b], Betul Uralcan[a,1,*]

[a]Department of Chemical Engineering, Bogazici University, Bebek, Istanbul, 34342, Turkey
[b]Science and Technology Research and Application Center (BITAM), Necmettin Erbakan University, Konya, 42090, Turkey

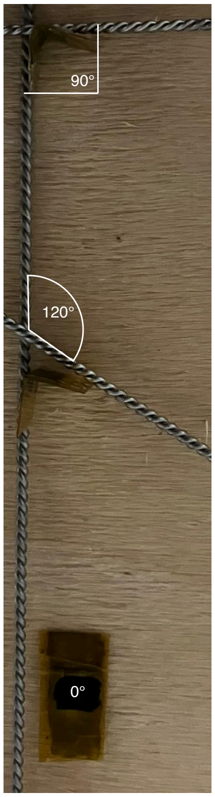

Figure S1: Flexible supercapacitor

*Corresponding author
Email address: betul.uralcan@boun.edu.tr (Betul Uralcan)

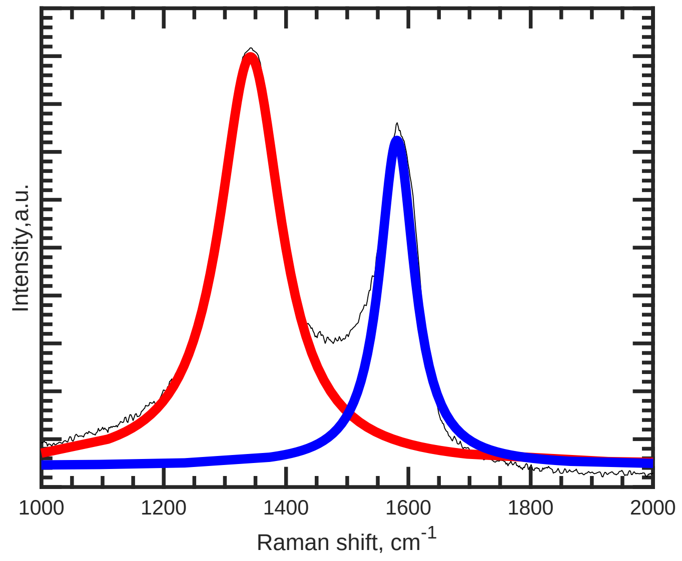

Figure S2: Raman analysis of BRGO

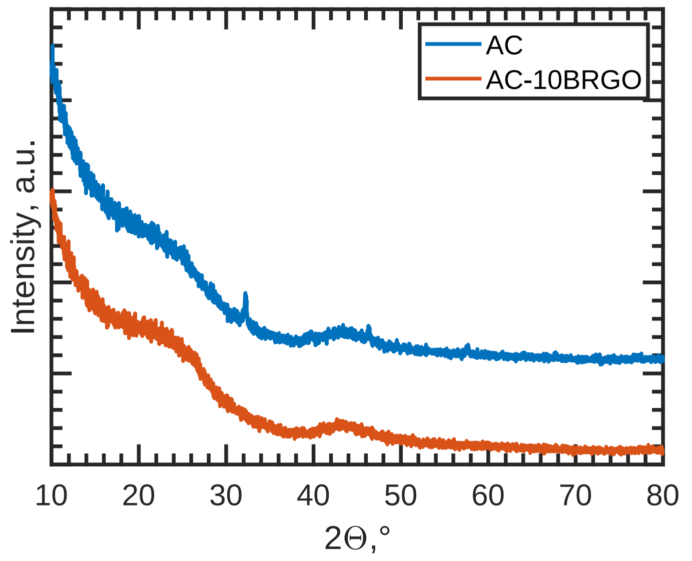

Figure S3: XRD spectrum of AC and AC-10BRGO

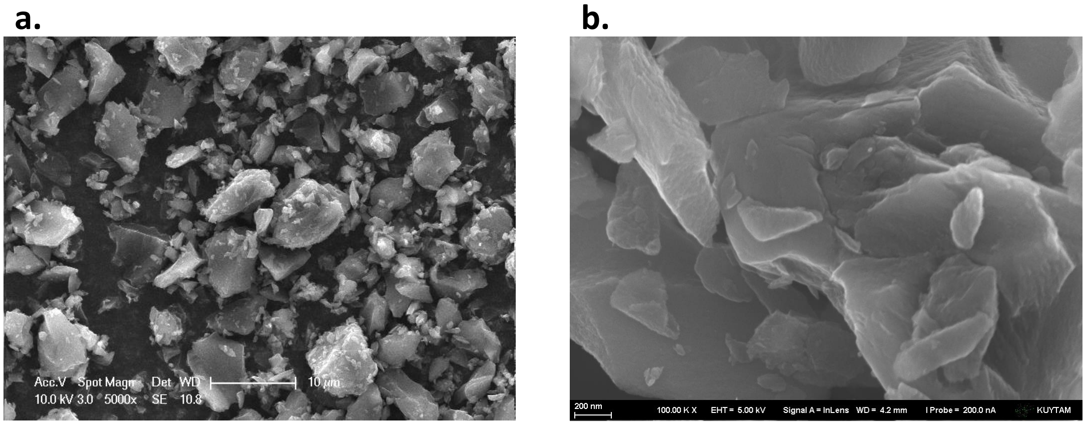

Figure S4: SEM images of a. AC and b. AC-10BRGO

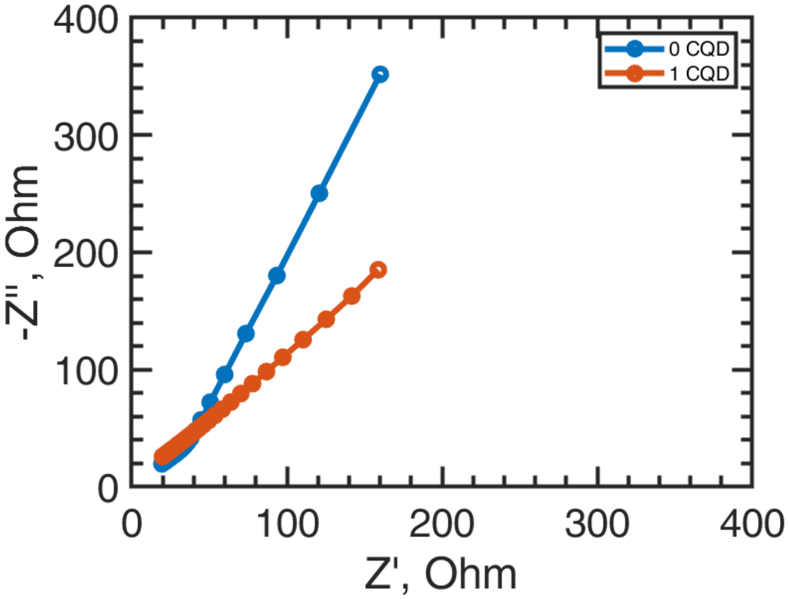

Figure S5: Impedance spectroscopy of AC-10BRGO and AC-10BRGO/1CQD

Table S1: A comparison of solid state supercapacitors with PVA-based gel electrolytes

Electrode	Electrolyte	Capacitance	Energy density, Wh/kg	Power density, W/kg	Cycling stability	Ref
AC	HQ-PVA/KOH	320 (F/g)	45	690	84.2 % over 1000 cycles	Jinisha et al., 2019
AC	KI-PVA/KOH	230 (F/g)	7.8	15000	96 % over 1000 cycles	Yu et al., 2011
AC	PVA/KOH	280 (F/g)	14.8	200	93 % over 3000 cycles	Jia et al., 2021
AC	PVA/KOH	190 (F/g)	4.8	10000	100 % over 1000 cycles	Barzegar et al., 2015
N,S doped CNS/Exfoliated graphene	PVA/KOH	200 (F/g)	6.3	2400	99 % over 10,000 cycles	Liu et al., 2018
N doped AC	PVA/KOH	270 (F/g)	7.9	250	90 % over 1000 cycles	Liu et al., 2019
AC	PVA/KOH	55 (F/g)	20	11000	80 % over 1000 cycles	Ponce et al., 2022
Graphene	PVA/H2SO4	30 ($\mu F/cm^2$)	7	1200	NA	Singh et al., 2016
AC	PVA/KOH	110 (F/g)	12.	900	80 % over 5000 cycles	Tian et al., 2021
N doped AC	PVA/KOH	250 (F/g)	16	100	70 % over 5000 cycles	Wang et al., 2016
Graphene	Cement-PVA/KOH	10 (F/g)	1.5	2250	NA	Xu and Zhang, 2020
Graphite	PVA/KOH	250 (F/g)	9	57	NA	Yang et al., 2021
AC-BRGO	CQD-PVA/KOH	107 (F/g)	29	875	90 % over 1000 cycles	This work
