# Supplementary figures and images for: Enhanced flexible supercapacitors with boron-doped graphene electrodes and carbon quantum dot gel electrolytes
Source: RSC Adv. 2025 Feb 14;15(7):5011–9. doi: 10.1039/d4ra06990k (PMC11827676; doi:10.1039/d4ra06990k)

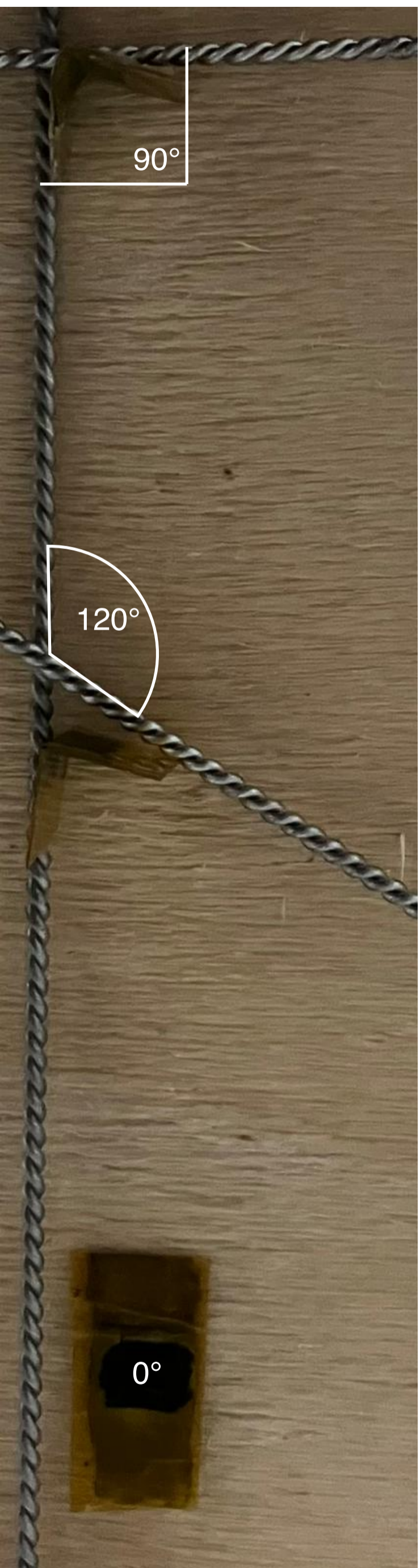

Supplement: RA-015-D4RA06990K-s003 [file RA-015-D4RA06990K-s003.pdf]

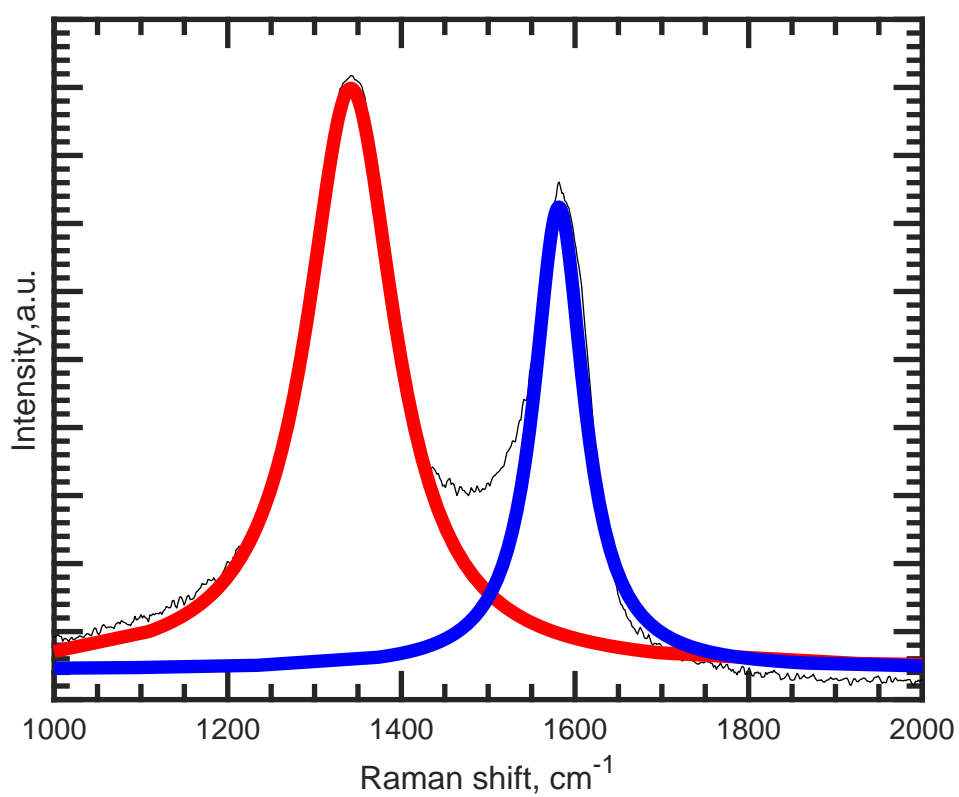

Supplement: RA-015-D4RA06990K-s004 [file RA-015-D4RA06990K-s004.pdf]

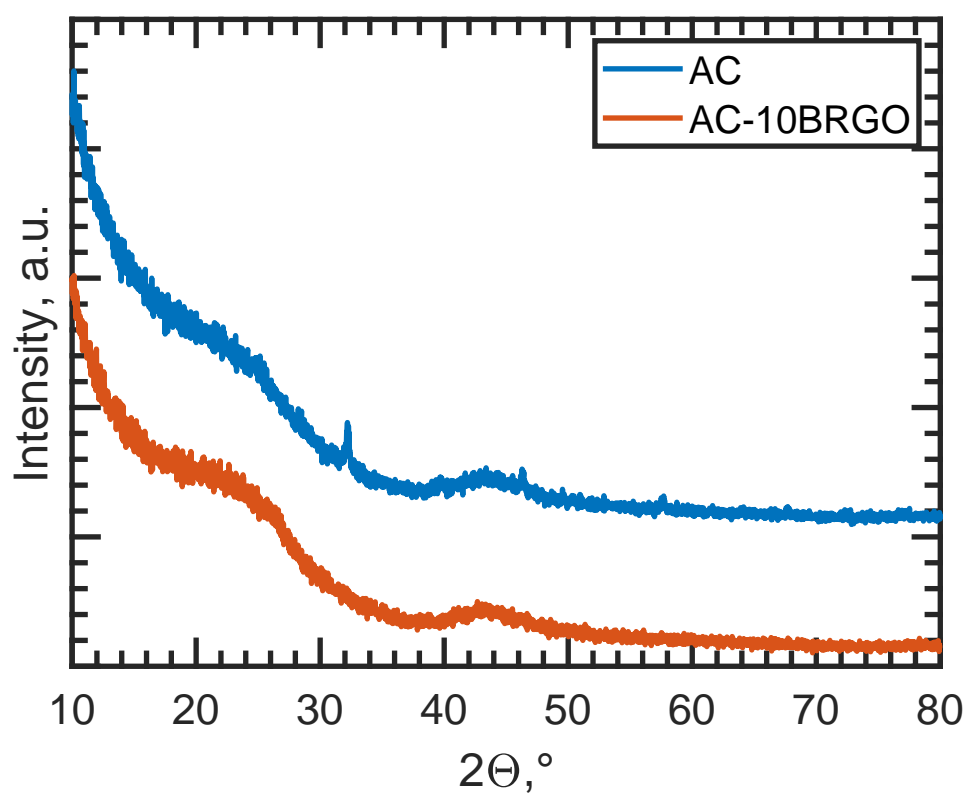

Supplement: RA-015-D4RA06990K-s005 [file RA-015-D4RA06990K-s005.pdf]

**a.**

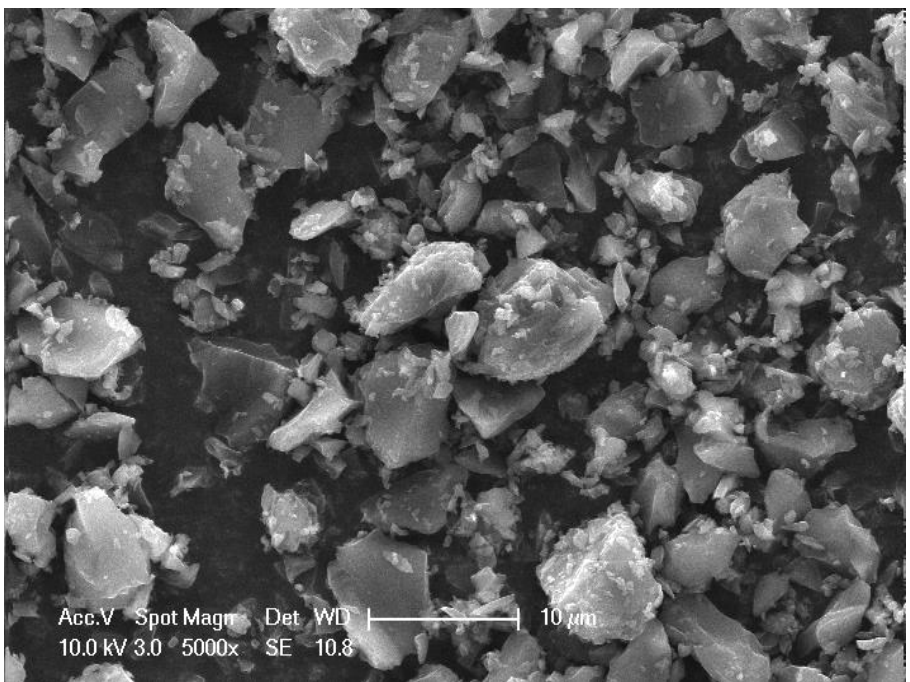

**b.**

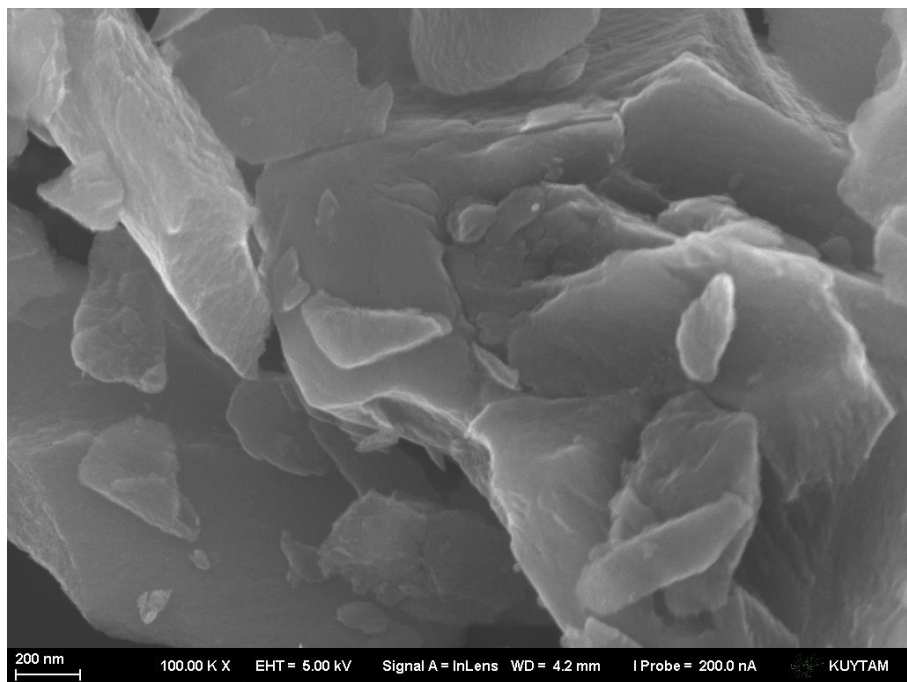

Supplement: RA-015-D4RA06990K-s006 [file RA-015-D4RA06990K-s006.pdf]

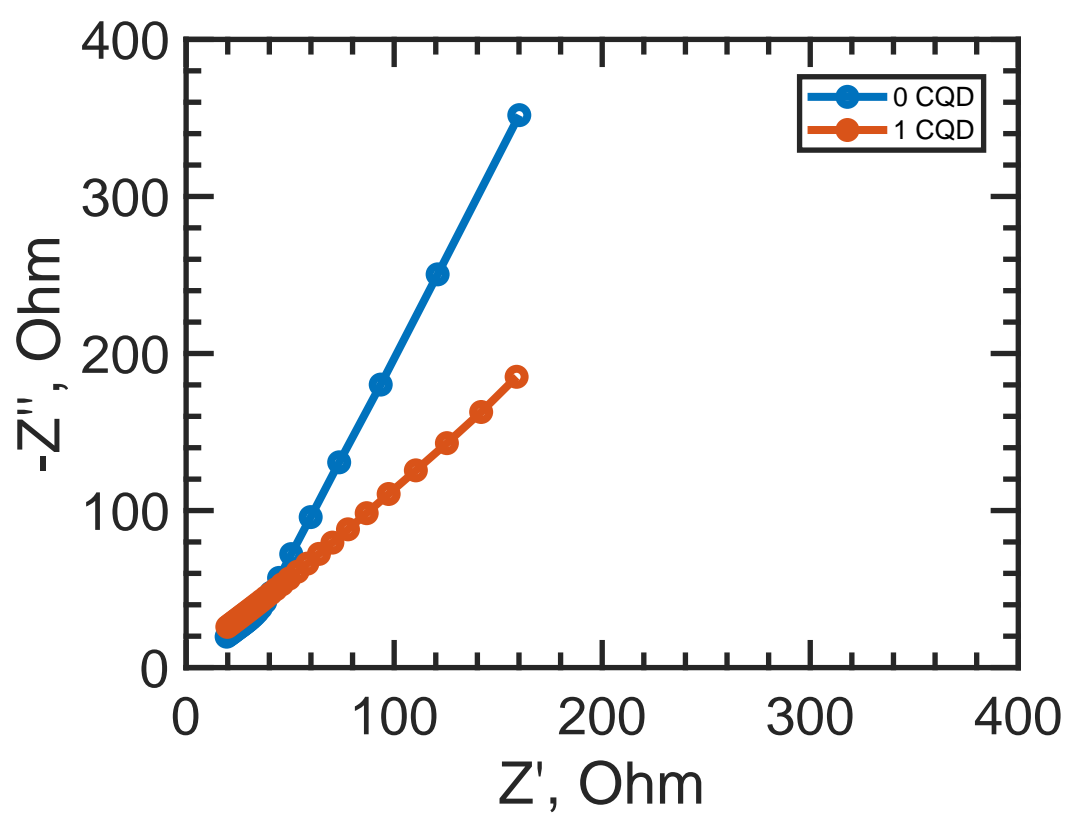

Supplement: RA-015-D4RA06990K-s007 [file RA-015-D4RA06990K-s007.pdf]
